# Monkeypox in a patient with HIV: case report

**DOI:** 10.17843/rpmesp.2023.402.12344

**Published:** 2023-05-10

**Authors:** Alex Omar Franco Lacato, Nataniel Aldo Chaparro Mérida, Dayany Moreno Samper, Delmira Selmira Orellana Padilla, Daniel Vides Melendres, Jhossmar Cristians Auza Santivañez

**Affiliations:** 1 San Juan de Dios University Hospital, Santa Cruz de la Sierra, Bolivia. San Juan de Dios University Hospital Santa Cruz de la Sierra Bolivia; 2 PROSALUD, Cochabamba, Bolivia. PROSALUD Cochabamba Bolivia; 3 Salvador Allende Clinical and Surgical Teaching Hospital, La Habana, Cuba. HSalvador Allende Clinical and Surgical Teaching Hospital La Habana Cuba; 4 Fray Quebracho Hospital, Tarija, Bolivia. Fray Quebracho Hospital Tarija Bolivia; 5 Franz Tamayo University, Santa Cruz de la Sierra, Bolivia. Franz Tamayo University Franz Tamayo University Santa Cruz de la Sierra Bolivia

**Keywords:** Monkeypox, HIV, Syphilis, Gonorrhea, Monkeypox Virus, Proctitis, Bolivia

## Abstract

Monkeypox (Mpox) is a zoonotic disease, endemic in some areas of Africa. But since May 2022, multiple cases of Mpox have been reported in non-endemic countries. We present the case of a patient with a history of HIV, as well as rash in several areas of the body, mostly in the gluteal region, associated with cervical lymphadenopathy and infectious proctitis. Diagnosis was confirmed by real-time polymerase chain reaction (RT-PCR) of skin lesion samples. Treponema *pallidum* and *Neisseria gonorrhoeae* infection was confirmed by serology and rectal discharge culture, respectively. The patient received antibiotics specific for gonorrhea and syphilis and his condition improved due to symptomatic and immunomodulatory therapy.

## INTRODUCTION

Monkeypox (mpox) is an emerging zoonotic disease caused by the monkeypox virus (MPXV), a member of the genus *Orthopoxvirus* from the family *Poxviridae*. MPXV is one of four *Orthopoxvirus* species that affect humans, along with variola virus, which causes smallpox, now eradicated; bovine smallpox virus; and vaccinia virus ^1^.

Previous outbreaks occurred mainly in Central and West Africa, primarily in the Congo Basin, however, it is no longer limited to those regions ^2^^,^^3^. On July 23, 2022, the Director-General of the World Health Organization declared the growing global outbreak of mpox a public health emergency of international concern (PHEIC) ^4^. The clinical manifestations are similar to those of smallpox, but the disease is milder and can cause high fever, headache, lymphadenopathy, exanthema, and enanthema; in previous outbreaks the case fatality rate was about 1% to 10%^3^.

This zoonotic disease has been reported in several countries and as of March 16, 2023, 86,500 laboratory-confirmed cases have been reported worldwide. To date, the crude case-fatality rate of this outbreak is approximately 0.0013%. However, 111 deaths have been officially reported and were mainly attributed to the underlying diseases associated with this disease^5^^,^^6^.

Mpox is an endemic disease mainly found in tropical rainforest areas of West and Central Africa ^7^^,^^8^. The clade of the Congo Basin (Central Africa) has the highest virulence. Some factors related to poor prognostic are parasitic co-infections and immunosuppressive conditions such as malnutrition and primary immunodeficiencies, HIV/AIDS, leukemias, lymphomas, solid organ transplantation and immunosuppressive therapies that affect the progression of the patient with mpox ^7^.

This disease predominantly affects homosexual and bisexual men between 20 and 50 years of age ^5^^,^^9^. It can be spread by sexual intercourse, through direct contact with the infected rash ^9^.

The information on mpox associated with HIV and sexually transmitted infections is scarce; this information could allow understanding the disease progression and prognosis, as well as the use of immunomodulatory therapy as a complementary treatment. 

We present the case of a male patient with mpox associated with sexually transmitted infections, who had HIV as an underlying condition.

## CASE REPORT

Male patient, 30 years old, resident of the city of Santa Cruz de la Sierra in Bolivia, engineer, man who has sex with man (MSM), promiscuous, diagnosed seven years ago with HIV, and with adherence to highly active antiretroviral therapy (HAART, dolutegravir, lamivudine and tenofovir), since the beginning of 2018.

On August 8, 2022, the patient reported unprotected sexual activity (without a condom) with a stranger, after four days he presented a papule with well-defined borders in the left gluteal region, near the intergluteal cleft and three centimeters from the gluteal sulcus (first day, August 12), with pruritus. In addition, he presented rectal tenesmus, non-fetid mucopurulent secretion and painful defecation. For this reason, he decided to go to the National Center for Tropical Diseases (CENETROP) on August 15, 2022.

**Figure 1:**
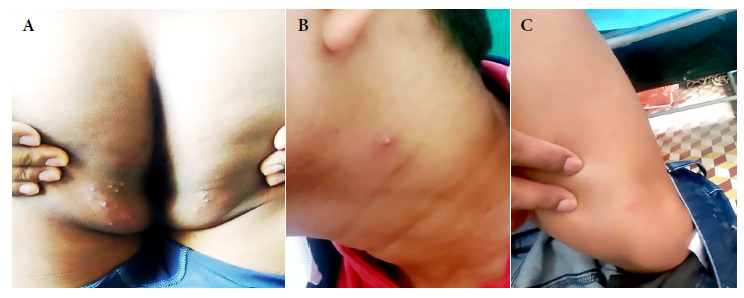
Third day. Skin lesions (polymorphic). (A) Papular, vesicular and pustular lesions (fifteen). (B) A slightly erythematous pustule on the left side of the neck. (C) Hardly visible papule with an erythematous halo.

Physical examination (third day) revealed 15 lesions, including papules and pustules smaller than one cm, located in the gluteal region. In addition, an erythematous papule was found in the lower third of the left thigh (sartorius) as well as a pustule in the neck and superficial and deep, bilateral, symmetrical, painless, mobile and slightly indurated cervical swollen lymph glands (Figure 1). Samples from skin lesions were obtained by pharyngeal and anal swabs, the latter to rule out sexually transmitted infections. Mpox diagnosis was confirmed by real-time polymerase chain reaction (RT-PCR). The patient was referred to a healthcare center; in addition, blood and serological tests, among others, were performed (Table 1).

**Table 1:** Laboratory results from a patient with monkeypox, HIV, gonorrhea and latent syphilis. Santa Cruz de la Sierra, Bolivia.

Test	Before monkeypox (09-08-22)	Third day (15-08-22)	Ninth day (17-08-22)	Twenty-fifth day
T CD4+ Lymphocytes	447 cel/μl	-	-	408 cel/μl
HIV viral load	Undetectable	-	-	Undetectable
RT-PCR for MPXV	-	Positive	-	-
Leucocytes	-	5.3 x 10^3^/μL	-	4.97 x 10^3^/μL
Stabs	-	0%	-	0%
Segmented	-	30%	-	44%
Eosinophiles	-	0%	-	4%
Lymphocytes	-	70%	-	52%
Red blood cells	-	5.488 x 10/μL	-	5.488 x 10/μL
Hematocrit	-	49%	-	43%
Hemoglobin	-	16.2 g/dL	-	15.8 g/dL
Platelets	-	302,000 mm^3^	-	223,000 mm^3^
Glucose	-	88 mg/dL	-	90 mg/dL
Creatinine	-	1.2 mg/dL	-	1.2 mg/dL
Urea	-	39 mg/dL	-	34 mg/dL
Glutamic pyruvic transaminase	-	29 U/L	-	29 U/L
Glutamic oxaloacetic transaminase	-	24 U/L	-	22 U/L
Alkaline phosphatase	-	181 UI/L	-	181 UI/L
C-reactive protein	-	Less than 6 mg/L	-	Less than 6 mg/L
Indirect hemagglutination for toxoplasmosis	-	Negative	-	Negative
VDRL	-	1/8	-	-
FTA-Abs	-	-	Positive	-
Culture of anal secretion and antibiogram	-	*Neisseria gonorrhoeae* was isolated at 48 hours and at 72 hours it was found to be sensitive to ceftriaxone.	-	-

RT-PCR: real-time polymerase chain reaction. VDRL: Venereal Disease Research Laboratory. FTA-Abs: fluorescent treponemal antibody absorption test. cell/μl: cells/microliters.

**Figure 2:**
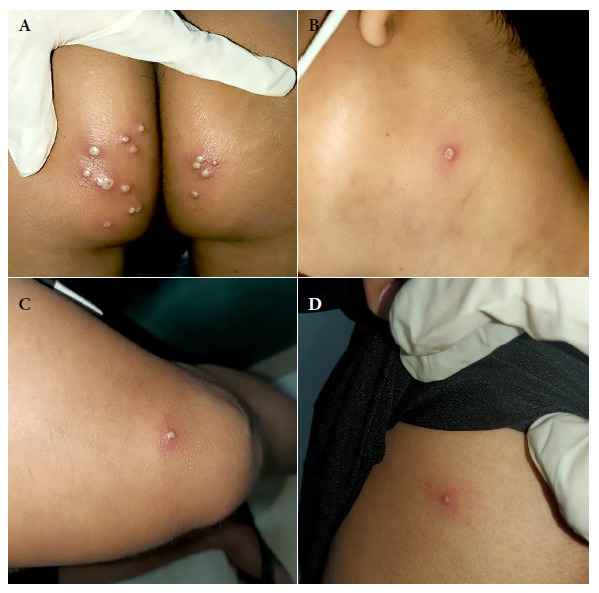
Tenth day. Skin lesions: (A) Pustular lesions in the buttock, some confluent and umbilicated. (B) An umbilicated pustule on the left side of the neck. (C) Pustule with an erythematous halo at the level of the sartorius muscle. (D) An erythematous papule in the left costal grill.

**Figure 3:**
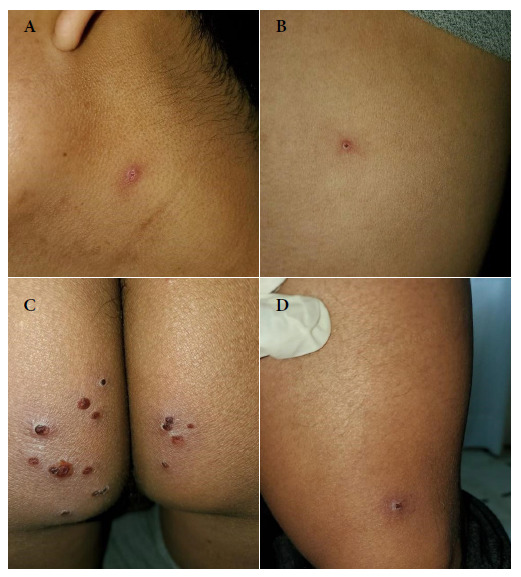
Fourteenth day. Skin lesions: (A) Scab fell from the neck (B) Scab in the left costal grid (C) Scab-like lesions at the gluteal level (D) Scab at the level of the sartorius muscle.

**Figure 4:**
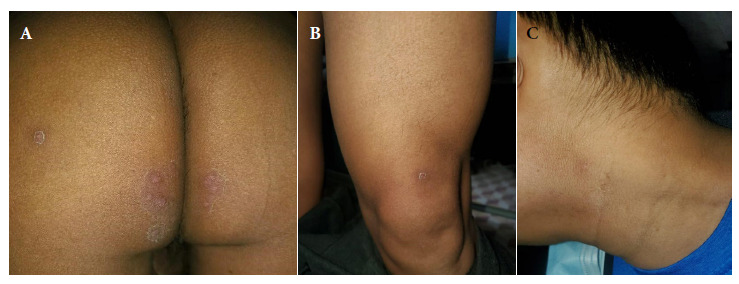
Twenty-first day. Scabs fell from (A) Gluteus (B) Sartorius (C) Neck.

Two solitary lesions appeared on the seventh day: a pustule on the left lateral region of the neck and an erythematous papule on the left costal grill. On the eleventh day, all the lesions in the gluteal region and neck were erythematous-pustular, and some pustules were umbilicated. In addition, the patient mentioned that the lesions in the gluteal region were painful (Figure 2). Most lesions were crusted and painless on the fourteenth day. A papule appeared near the gluteal cleft (Figure 3). On the eighteenth day, several crusts fell off. On the twenty-first day all the scabs fell off and the patient was discharged (Figure 4).

In view of these findings, the following conditions were considered: mpox with HIV, gonorrhea, latent syphilis and infectious proctitis (IP). The following measures were applied from the fifth day onwards: home isolation, frequent disinfection, daily washing of towels, clothing and bedding at a temperature of 60°C, drying of the lesions with a specific towel for the affected areas, and a different one for the rest of the body. The patient received empirical and symptomatic pharmacological treatment, which included: ceftriaxone 1g single intramuscular (IM) dose; penicillin G benzathine 2.4 million IU once a week for three weeks IM; transfer factor (HEBERTRANS®) twice a week IM for eight weeks; doxycycline 100 mg twice a day orally for seven days; vitamin D3 150,000 IU single dose orally (PO); vitamin C 1g every eight hours PO; fexofenadine one 180 mg tablet once a day PO; tramadol 325mg/paracetamol 37.5mg one tablet three times a day PO; Roydil (calcium dobesilate, lidocaine anhydrous, hydrocortisone acetate and zinc oxide) once a day rectally. Tenesmus improved three days after using ceftriaxone.

## DISCUSSION

In a multinational study, Thornhill JP *et al*. reported 528 mpox cases (527 men and one woman) in 16 countries between April to June 2022. The median age was 38 years (range 18 to 68 years), showing that mpox disproportionately affected the MSM population, suggesting an increase of transmission through sexual networks ^10^. 

There are several poor prognostic factors for mpox, both virus- and host-dependent. Two distinct clades of MPXV have been identified: Central Africa (also known as Congo Basin) and West Africa. The Central African clade is more virulent, with an average mortality rate of 10.6%, compared with 3.6% for the West African clade. All cases reported outside Africa, including those currently circulating, have been caused by the West African clade ^11^.

Severe infection is more frequent among vulnerable populations, such as infants, pregnant women, people with primary or secondary immunodeficiency, particularly those with HIV/AIDS, poor virological (detectable viral load greater than 200 copies/μL) and immunological (CD4+ T lymphocytes less than 200 cells/μL) control ^12^.

Patel A. *et al*., in a study on 197 individuals, reported that approximately 32% also had a a sexually transmitted infection, and the most common co-infections found in rectal samples were *Neisseria gonorrhoeae* and *Chlamydia trachomatis*^13^. 

Acute IP is mostly found in the MSM population and the main causative agents are *Neisseria gonorrhoeae*, *Chlamydia trachomatis*, *Treponema pallidum* and herpes simplex virus; although MPXV should also be considered ^14^^,^^15^. In this case, according to the clinical findings, the infectious agents (*Neisseria gonorrhoeae* and MPXV) could have caused IP. However, the most common cause of IP is *Neisseria gonorrhoeae,* and its signs and symptoms were reported in this case; in addition, the response to treatment with ceftriaxone was good, which suggests that this microorganism may have been responsible; excluding *Treponema Pallidum* (primary syphilis), whose incubation period is usually from two to six weeks ^16^.

Mailhe *et al*., in a study that included 246 participants, reported that 99% were men, of whom 45 had anal and digestive complications and only one patient received cidofovir, an intravenous antiviral treatment for severe keratitis ^17^.

Several studies have shown that MPXV has a direct cytopathic effect on the cells of innate and adaptive immunity; in addition, these viruses inhibit the cytotoxic and antiviral function of natural killer (NK) cells, and reduce the activation of CD4+ and CD8+ T lymphocytes, which could complicate the prognosis of immunocompromised patients. MPXV is capable of stimulating NK cells, thus proliferating in peripheral blood and lymph nodes, causing multiple lymphadenopathies ^11^^,^^18^.

Although, the patients’ CD4+ T lymphocytes count was greater than 200 cells/μL, which was associated with a rapid spread of skin lesions in various body regions, an immunostimulant was used to improve the immune status. Transfer factor (TF) is a blood product consisting of a dialyzable extract of leukocytes that transfers immunity from donors to an immune-deficient recipient. It is mainly used in patients with cellular immunodeficiency and viral infections. It is registered as a drug in Cuba and is produced by the Centro de Ingeniería Genética y Biotecnología, under the trade name Hebertrans® ^19^.

One of the limitations of this report is that the patient did not receive any antiviral drugs such as tecovirimat, brincidofovir and cidofovir, since they are not available in Bolivia. Countries should invest in vaccines and in the treatment of mpox in order to face this zoonosis.

Currently, there is no specific or approved treatment for mpox, so therapy mainly aims at managing symptoms. However, some antivirals called “of compassionate use” have been approved by the Food and Drug Administration (FDA) for treatment of human smallpox and have shown efficacy against mpox; the Centers for Disease Control and Prevention (CDC) guidelines suggest tecovirimat as first-line drug in patients with severe disease or who are at high risk of developing severe disease ^16^.

Rectoscopy, nuclear magnetic resonance imaging and contrast tomography would have helped to determine the severity of IP; however, these tests were not performed due to the stigmatization of this zoonosis and the adequate disease progression ^20^.

In conclusion, the current mpox outbreak affects MSM, and causes dermatologic manifestations with polymorphic evolution, which may or may not be associated with IP. Due to the epidemiological context, MPXV should be considered during differential diagnosis in HIV seropositive patients with IP. Rectoscopy, imaging studies and molecular identification by RT-PCR are recommended for the correct diagnosis of IP by MPXV. Treatment aims to manage symptoms, although tecovirimat is recommended in severe cases.
