# Supplementary material for: Monkeypox in a patient with HIV: case report
Source: Rev Peru Med Exp Salud Publica. 2023 May 10;40(2):229–35. doi: 10.17843/rpmesp.2023.402.12344 (PMC10953656; doi:10.17843/rpmesp.2023.402.12344)
Supplement: Translation

# Translation: Viruela símica en un paciente con VIH: reporte de caso

**Authors:** Alex Omar Franco Lacato, Nataniel Aldo Chaparro Mérida, Dayany Moreno Samper, Delmira Selmira Orellana Padilla, Daniel Vides Melendres, Jhossmar Cristians Auza Santivañez

**Affiliations:** 1 Hospital Universitario San Juan de Dios, Santa Cruz de la Sierra, Bolivia.; 2 PROSALUD, Cochabamba, Bolivia.; 3 Hospital Clínico Quirúrgico Docente «Salvador Allende», La Habana, Cuba.; 4 Hospital «Fray Quebracho», Tarija, Bolivia.; 5 Universidad Franz Tamayo, Santa Cruz de la Sierra, Bolivia.

**Keywords:** Viruela Símica, VIH, Sífilis, Gonorrea, Virus de la Viruela del Simio, Proctitis, Bolivia

## Abstract

La viruela símica es una enfermedad zoonótica endémica en algunas zonas de África; desde mayo del 2022 se confirmaron múltiples reportes de viruela símica (VS) en países no endémicos. Se presenta el caso de un paciente con antecedente de VIH con exantemas en diversas regiones del cuerpo, con mayor concentración en la región glútea, asociado a adenopatías cervicales y proctitis infecciosa. Se procedió a la toma de la muestra de las lesiones dermatológicas para el estudio molecular de reacción en cadena de la polimerasa en tiempo real (RT-PCR), que confirmó el diagnóstico de VS. La infección por *Treponema pallidum* y *Neisseria gonorrhoeae* se identificó mediante serología y cultivo de secreción anal, respectivamente. Se aplicó tratamiento con antibióticos específicos para gonorrea y sífilis, y la VS respondió favorablemente a la terapia sintomática e inmunomoduladora.

## INTRODUCCIÓN

La viruela símica (VS) es una enfermedad zoonótica emergente causada por el virus de la viruela del simio (VVS), un miembro del género *Orthopoxvirus* en la familia *Poxviridae*. La VVS es una de las cuatro especies de Orthopoxvirus patógenas para humanos, junto con el virus variola, el agente causal de la viruela, ahora erradicado en la naturaleza; virus de la viruela bovina y virus vaccinia ^1^.

Los brotes anteriores ocurrieron principalmente en África Central y Occidental, principalmente en la cuenca del Congo, sin embargo, ya no se limita a esas regiones ^2^^,^^3^. El 23 de julio del 2022, el director general de la Organización Mundial de la Salud declaró al creciente brote global de la VS como una emergencia de salud pública de preocupación internacional (PHEIC, por sus siglas en inglés) ^(^^4^. Las manifestaciones clínicas son similares a los de la viruela, pero la enfermedad es más leve y se manifiesta principalmente con fiebre alta, cefalea, linfadenopatía, exantema y enantema; en los anteriores brotes la tasa de letalidad fue de alrededor del 1% al 10% ^3^.

Esta enfermedad zoonótica se ha reportado en diversos países y hasta el 16 marzo del 2023 se han notificado 86 500 casos confirmados por laboratorio a nivel mundial. La tasa bruta de letalidad de este brote, hasta la fecha, es de aproximadamente de 0,0013%. Sin embargo, se han notificado oficialmente 111 muertes, las cuales fueron atribuidas, principalmente, a las enfermedades de base asociadas a esta patología ^5^^,^^6^.

La VS es una enfermedad endémica que ocurre principalmente en áreas de la selva tropical de África Occidental y Central ^7^^,^^8^. El clado de la cuenca del Congo (África Central) presenta la mayor virulencia. Existen factores de mal pronóstico como las coinfecciones parasitarias y las condiciones de inmunodepresión como la desnutrición y las inmunodeficiencias primarias, VIH/sida, leucemias, linfomas, trasplante de órganos sólidos y terapias inmunosupresoras que afectan la evolución clínica del paciente con VS ^(^^7^.

Entre los casos reportados hasta este momento, se ha observado que esta enfermedad afecta predominantemente a hombres homosexuales y bisexuales, entre 20 a 50 años ^5^^,^^9^. Esta propagación puede darse por relaciones sexuales, a través del contacto directo con la erupción infectada ^9^.

Existe escasa información en la literatura científica sobre la VS asociada a VIH e infecciones de transmisión sexual, que permitan comprender la evolución clínica y pronóstico en los individuos afectados, así como el empleo de la terapia inmunomoduladora, como tratamiento complementario.

Se presenta el caso de un paciente varón con diagnóstico de VS, que tiene VIH como patología de base, asociado a infecciones de transmisión sexual.

## REPORTE DE CASO

Paciente varón de 30 años, residente de la ciudad de Santa Cruz de la Sierra en Bolivia, ingeniero, hombre que tiene sexo con hombre (HSH), promiscuo y con antecedentes patológicos personales de infección por VIH, diagnosticado hace siete años, con adherencia a la terapia antirretroviral de gran actividad (TARGA, dolutegravir, lamivudina y tenofovir), desde inicios del 2018.

El 8 de agosto del 2022 refirió actividad sexual desprotegida (sin preservativo) con un desconocido, luego de cuatro días presentó una pápula con bordes bien definidos en la región glútea izquierda, cerca de la hendidura interglútea y a tres centímetros del surco glúteo (primer día, 12 de agosto), con prurito. Además, presentó pujo y tenesmo rectal, con secreción mucopurelenta no fétida y dolor a la defecación. Por tal razón decidió acudir al Centro Nacional de Enfermedades Tropicales (CENETROP) el 15 de agosto del 2022.

En el examen físico (tercer día) se observó 15 lesiones, entre pápulas y pústulas, localizadas en la región glútea de tamaños variables, menores a un cm. Además, se identificó una pápula eritematosa a nivel del tercio inferior del muslo izquierdo (sartorio) y una pústula a nivel del cuello, también se observaron adenopatías cervicales superficiales y profundas, bilaterales, simétricas, indoloras, móviles y ligeramente induradas (Figura 1). Se procedió a la toma de muestra de las lesiones dermatológicas, con hisopado faríngeo y anal, esta última para descartar infecciones de transmisión sexual. Se confirmó el diagnóstico de VS mediante reacción en cadena de la polimerasa en tiempo real (RT-PCR). Es referido a su centro de salud correspondiente; además, se realizaron pruebas de química sanguínea, serológicas y entre otras (Tabla 1).

**Figura 1:** Tercer día. Lesiones dermatológicas (polimórficas). (A) Lesiones de tipo papular, vesicular y pustulosas (quince). (B) Una pústula ligeramen-te eritematosa en la parte lateral izquierda del cuello. (C) Pápula de difícil observación con un halo eritematoso.

**Tabla 1:** Resultados de laboratorio de un paciente con diagnóstico de viruela símica con VIH y gonorrea y sífilis latente procedente de Santa Cruz de la Sierra en Bolivia.

Tipo de examen	Antes de la viruela símica (09-08-22)	Tercer día (15-08-22)	Noveno día (17-08-22)	Vigésimo quinto día
Cuantificación de linfocitos T CD4+	447 cel/μl	-	-	408 cel/μl
Carga viral para VIH	Indetectable	-	-	Indetectable
RT-PCR para VVS	-	Positivo	-	-
Leucocitos	-	5,3 x 10^3^/μL	-	4,97 x 10^3^/μL
Cayados	-	0%	-	0%
Segmentados	-	30%	-	44%
Eosinófilos	-	0%	-	4%
Linfocitos	-	70%	-	52%
Hematíes	-	5,488 x 10/μL	-	5,488 x 10/μL
Hematocrito	-	49%	-	43%
Hemoglobina	-	16,2 g/dL	-	15,8 g/dL
Plaquetas	-	302 000 mm^3^	-	223 000 mm^3^
Glucosa	-	88 mg/dL	-	90 mg/dL
Creatinina	-	1,2 mg/dL	-	1,2 mg/dL
Urea	-	39 mg/dL	-	34 mg/dL
Transaminasa glutámica pirúvica	-	29 U/L	-	29 U/L
Transaminasa glutámica oxalacética	-	24 U/L	-	22 U/L
Fosfatasa alcalina	-	181 UI/L	-	181 UI/L
Proteína C reactiva	-	Menor a 6 mg/L	-	Menor a 6 mg/L
Hemaglutinación indirecta para toxoplasmosis	-	Negativo	-	Negativo
VDRL	-	1/8	-	-
FTA-Abs	-	-	Positivo	-
Cultivo de secreción anal más antibiograma	-	Se aísla *Neisseria gonorrhoeae* a las 48 horas y a las 72 horas se determina que es sensible a ceftriaxona	-	-

RT-PCR: reaccion en cadena de la polimerasa en tiempo real, VDRL: venereal disease research laboratory, FTA-Abs: anticuerpos absorbidos fluorescentes antitreponema, VVS: virus de la viruela del simio.

Se decidió aislamiento y vigilancia domiciliaria dos veces por semana (lunes y jueves), al séptimo día aparecen dos lesiones solitarias: una pústula en la región lateral izquierda del cuello y una pápula eritematosa en la parrilla costal izquierda. El décimo primer día, todas las lesiones son eritemato-pustulosas en la región glútea y en cuello, y algunas pústulas se observaron umbilicadas. Además, el paciente refiere que las lesiones de la región glútea son dolorosas (Figura 2). El décimo cuarto día, la mayoría de las lesiones están en fase de costra y sin dolor. Una pápula aparece cerca de la hendidura glútea (Figura 3). En el décimo octavo día se constató la caída de varias costras. En el vigésimo primer día caen todas las costras y se decide su alta (Figura 4).

**Figura 2:** Décimo día. Lesiones dermatológicas. (A) Lesiones pustulosas a nivel del glúteo, algunas confluentes y umbili-cadas. (B) Una pústula umbilicada en la parte lateral izquierda de cuello. (C) Pústula con un halo eritematoso a nivel del músculo sartorio. (D) Una pápula eritematosa a nivel de la parrilla costal izquierda.

**Figura 3:** Décimo cuarto día. Lesiones dermatológicas. (A) Costra caída a nivel del cuello. (B) Una costra a nivel de la parrilla costal izquierda. (C) Lesiones de tipo costra a nivel del glúteo. (D) Una costra a nivel del músculo sartorio.

**Figura 4:** Vigésimo primer día. Caída de las costras. (A) Glúteo. (B) Sartorio. (C) Cuello.ABCREFERENCIAS BIBLIOGRÁFICAS

Ante estos hallazgos, se platearon las siguientes entidades nosológicas: VS con VIH, gonorrea, sífilis latente y proctitis infecciosa (PI). A partir del quinto día, se indicaron medidas generales como aislamiento domiciliario, desinfección frecuente, lavado diario de toallas, ropa de vestir y cama a una temperatura de 60°C, secado de las lesiones con una toalla específica para las zonas afectadas, y otra para el resto del cuerpo. Se indicó tratamiento farmacológico empírico y sintomático con ceftriaxona 1g dosis única vía intramuscular (IM); penicilina G benzatínica 2.4 millones UI, una vez por semana durante tres semanas por vía IM; factor de transferencia (HEBERTRANS®) dos veces por semana por vía IM durante ocho semanas; doxiciclina 100 mg dos veces al día vía oral (VO) durante siete días; vitamina D3 150 000 UI dosis única por VO; vitamina C 1 g cada ocho horas VO; fexofenadina un comprimido de 180 mg una vez al día VO; tramadol 325 mg/paracetamol 37,5mg un comprimido tres veces al día VO; Roydil (dobesilato de calcio, lidocaína anhídra, hidrocortisona acetato y óxido de zinc) una vez al día por vía rectal. Luego de tres días de la aplicación de la ceftriaxona, el paciente tuvo mejoría del pujo y tenesmo.

## DISCUSIÓN

El estudio multinacional realizado por Thornhill *et al*. reportó 528 casos de VS (527 hombres y una mujer) en 16 países, entre abril y junio del 2022. La mediana de edad de los pacientes fue de 38 años (rango 18 a 68 años), indicando que la propagación actual ha afectado de manera desproporcionada a la población HSH, lo que sugiere una amplificación de la transmisión a través de redes sexuales ^10^.

Existen diversos factores de mal pronóstico de la VS, dependiente del virus y del hospedero. Se han identificado dos clados distintos del VVS: África Central (también conocida como Cuenca del Congo) y África Occidental. El clado de África Central es más virulento, con una tasa de mortalidad promedio del 10,6%, en comparación con el 3,6% para el clado de África Occidental. Todos los casos notificados fuera de África, incluidos los que están actualmente en circulación, han sido causados ​​por el clado de África Occidental ^11^.

Las poblaciones vulnerables que evolucionan a una infección grave son los infantes, mujeres embarazadas, personas con inmunodeficiencia, ya sea de causa primaria o secundaria, sobre todo individuos con VIH/SIDA, con un mal control virológico (carga viral detectable mayor a 200 copias/µL) e inmunológico (linfocitos T CD4+ menor a 200 cel/µL) ^12^.

La investigación realizada por Patel *et al*. que incluyó 197 individuos, encontró que el 32%, aproximadamente, concomitaban con una infección de transmisión sexual y las coinfecciones más comunes fueron por *Neisseria gonorrhoeae* y *Chlmydia trachomatis* en la muestra rectal ^13^.

La PI aguda se observa con mayor frecuencia en la población HSH y los principales patógenos responsables son *Neisseria gonorrhoeae*, *Chlamydia trachomatis*, *Treponema pallidum* y el virus herpes simple; aunque también se debe considerar al VVS ^14^^,^^15^. En este caso, de acuerdo a los hallazgos clínicos, se podría inferir que los agentes infecciosos (*Neisseria gonorrhoeae* y VVS*)* son causantes de la PI. Sin embargo, la causa más común de PI es la *Neisseria gonorrhoeae*, cuya clínica es compatible con el presente caso; además, se observó buena respuesta a la administración de ceftriaxona, lo que sugiere como responsable a este agente infeccioso; excluyendo al *Treponema Pallidum* (sífilis primaria) debido a que el período de incubación es de dos a seis semanas ^16^^).^

En estudio realizado por Mailhe *et al*. que incluyó 246 individuos, describió que el 99% eran varones, de los cuales 45 mostraron complicaciones anales y digestivas y solo un paciente recibió tratamiento antiviral intravenoso, cidofovir por queratitis severa ^17^.

En diversos estudios se demostró que el VVS tiene un efecto citopático directo en las células de la inmunidad innata y adaptativa, además estos virus inhiben la función citotóxica y antiviral de las células *natural killer* (NK), y reducen la activación de linfocitos T CD4+ y CD8+ lo que podría complicar la evolución clínica en los pacientes inmunocomprometidos. Se evidenció que el VVS es capaz de estimular a las NK, de esta forma proliferan en la sangre periférica y en los ganglios linfáticos, ocasionando múltiples linfadenopatías ^11^^,^^18^.

Aunque, el paciente presentó valores absolutos de linfocitos T CD4+ mayor a 200 cel/µL, asociado a una rápida propagación de las lesiones cutáneas en diversas regiones corporales, se decidió administrar un inmunoestimulante para mejorar el estado inmunológico. El factor de transferencia (FT) es un hemoderivado que consiste en un extracto dializable de leucocitos que transfiere inmunidad de varios donantes a un receptor con déficit inmunológico. Su principal indicación es en pacientes con inmunodeficiencia celular e infecciones virales. En Cuba está registrado como medicamento, y lo produce el Centro de Ingeniería Genética y Biotecnología, con el nombre comercial de Hebertrans® ^19^.

Como limitaciones del presente reporte se debe mencionar que al paciente no se le administró ningún fármaco antiviral como tecovirimat, brincidofovir y cidofovir, ya que no están disponibles en Bolivia. Los países deben invertir en vacunas y en el tratamiento de la VS, con la finalidad de enfrentar a esta zoonosis.

Actualmente, no existe un tratamiento específico y aprobado para la VS, por lo que su manejo es sintomático. Sin embargo, existen antivirales que han sido aprobados, denominados de «uso compasivo» por la Administración de Alimentos y Medicamentos (FDA, por sus siglas en inglés) para viruela humana y han demostrado eficacia contra la VS; la guía de los Centros para el Control y Prevención de Enfermedades (CDC, por sus siglas en inglés) sugieren al tecovirimat como uso de primera línea en pacientes con cuadros graves o que poseen alto riesgo de presentar un cuadro grave ^16^.

La rectoscopia, la resonancia magnética nuclear y la tomografía con contraste hubieran determinado la gravedad de la PI; sin embargo, debido a la estigmatización de esta zoonosis y a la buena evolución clínica, no se realizaron estos estudios ^20^.

En conclusión, el brote actual de la VS afecta a los HSH, con manifestaciones dermatológicas con evolución polimórfica, que puede estar o no asociada a una PI. En pacientes VIH seropositivos con PI se sugiere el diagnóstico diferencial como posible agente causal al VVS enmarcado en la situación epidemiológica. Para el correcto diagnóstico de la PI ocasionada por VVS, se recomienda efectuar rectoscopia, estudios imagenológicos e identificación molecular mediante RT-PCR. El manejo es sintomático, aunque en casos severos se recomienda la administración de tecovirimat.
